# Deep learning for multiclass tumor cell detection in histopathology slides of hereditary diffuse gastric cancer

**DOI:** 10.1016/j.isci.2025.113064

**Published:** 2025-07-05

**Authors:** Robin Lomans, Valentina Angerilli, Joey Spronck, Liudmila L. Kodach, Irene Gullo, Fátima Carneiro, Rachel S. van der Post, Francesco Ciompi

**Affiliations:** 1 Department of Pathology, Radboud University Medical Centre, Nijmegen, the Netherlands; 2 Department of Pathology, The Netherlands Cancer Institute, Amsterdam, the Netherlands; 3 Department of Pathology, Unidade Local de Saúde São João, Porto, Portugal; 4 Department of Pathology, Faculty of Medicine of the University of Porto (FMUP), Porto, Portugal; 5 Instituto de Investigação E Inovação Em Saúde (i3S) & Institute of Molecular Pathology and Immunology of the University of Porto (Ipatimup), Porto, Portugal

**Keywords:** Cancer, Artificial intelligence

## Abstract

Hereditary diffuse gastric cancer (HDGC) is a rare condition where early tumor detection is challenging due to diffuse infiltration and tumor heterogeneity. Accurate identification of DGC cells is essential for understanding tumor behavior. This study aimed to develop deep learning models to automatically detect key tumor cell types—typical and atypical signet ring cells and non-signet ring tumor cells—in H&E-stained digital pathology slides from HDGC patients. Using a multi-center dataset of 350 whole-slide images and over 91,000 annotated cells from 43 patients, we trained nnU-Net models for cell detection and compared them to Faster R-CNN baselines. We also conducted a reader study with five pathologists to benchmark performance. nnU-Net outperformed both pathologist inter-observer agreement and Faster R-CNN, achieving an F_1_ score of 0.49. It also matched human-level performance in estimating lesion size and cell type distributions, demonstrating its potential to support DGC tumor detection and analysis.

## Highlights

• A multicentre dataset of 350 whole-slide images with 91,939 annotations was curated
• nnU-Net outperforms Faster R-CNN and pathologists on detecting three cell types
• Potential for AI-assisted diagnostics of hereditary diffuse gastric cancer patients
• Deep learning may aid understanding of tumor behavior in diffuse gastric cancer

## Graphical abstract

**Figure:**
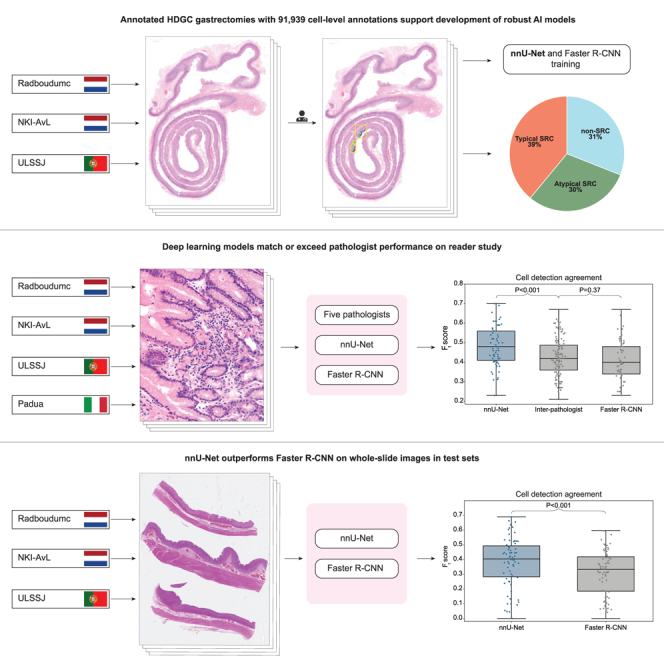

## Introduction

Diffuse gastric cancer (DGC) is a histological subtype of gastric cancer characterized by diffuse infiltration of poorly cohesive tumor cells through the gastric wall.^1^ A subset of DGC cases is hereditary (HDGC),^2^ with an estimated population incidence of 5–10/100,000 births,^3^ primarily caused by inactivation of the *CDH1* gene. Individuals at risk for HDGC are typically offered a prophylactic total gastrectomy (PTG), which, under total embedding protocol, generate over 100 histology slides per stomach.^3^ Alternatively, annual surveillance endoscopies produce over 15 slides per procedure. Both approaches place a considerable burden on pathology practices, requiring extensive effort for thorough examination and posing a risk of missing cancerous lesions.

In PTG specimens, early-stage T1a HDGC is commonly observed, characterized by well-differentiated signet ring cells (SRCs). This stage is generally considered indolent, with no associated or very low risk of metastatic disease.^4^^,^^5^ However, it has been recognized that a subset of early-stage HDGC exhibits atypical features, suggesting a transitional stage. These lesions remain confined to the mucosa (T1a), but display a different cellular composition, marked by fewer SRCs and an increase in smaller, irregular cells.^6^^,^^7^ Identifying the composition of early HDGC represents a new step in understanding tumor behavior, guiding surveillance endoscopy strategies, and informing prognostic assessments in PTG reports. However, consistent morphological assessment has not yet been introduced in clinical practice.^8^ Developing automated tools to detect and classify the various tumor cell types in DGC could assist pathologists in detecting these tumors more efficiently and accurately. Moreover, such tools could provide deeper insights into the disease’s progression and help refine patient management strategies.

In recent years, deep learning has emerged as a powerful tool for automated analysis of digital pathology images.^9^ The shift from conventional microscopy to digital pathology workflows has enabled the application of artificial intelligence (AI) tools to whole-slide images (WSIs) to support pathologists in diagnostics. Convolutional neural network (CNN)-based object detection models like Faster R-CNN have long served as state-of-the-art methods for locating cells in WSIs.^10^ Recently, segmentation methods, which perform pixel-wise classification, have also shown promising results for cell detection. For instance, the U-Net architecture was successfully used for lymphocyte detection in histopathology images,^11^ and segmentation methods were integral to the best performing solutions of the MIDOG21 and MIDOG22 challenges for mitosis detection.^12^^,^^13^^,^^14^

Conventional object detection and segmentation models like Faster R-CNN and U-Net require substantial input from researchers to fine-tune hyperparameters for optimal performance. The self-configuring framework nnU-Net simplifies this process by automatically adapting to the specific characteristics of the training data, *de facto* eliminating the need for manual adjustments.^15^ While nnU-Net has been widely used in the field of radiology and shown to achieve state-of-the-art results in tasks like tumor segmentation in CT and MRI scans,^16^ it has only recently been extended to histopathology data.^17^ Therefore, the use of nnU-Net in digital pathology remains largely unexplored, as well as its effectiveness in a cell detection framework.

The DigestPath challenge introduced a partially annotated dataset of colon and stomach images containing SRCs, which led to the development of several algorithms for SRC detection.^18^^,^^19^^,^^20^^,^^21^^,^^22^^,^^23^^,^^24^ However, the dataset and its derived algorithms were limited to cancers with SRCs and did not address other types of tumor cells, which are critical for understanding DGC tumor behavior. While subsequent research proposed methods to quantify the inherent morphological atypia of SRCs,^25^ no AI system has been developed to detect and categorize different tumor cell types in early DGC tumors.

In this study, we developed deep learning models to detect both SRCs and non-SRC tumor cells in H&E-stained PTG slides from patients with HDGC. SRCs were further classified as either typical or atypical based on adherence to established morphological criteria.^8^ To achieve this, we assembled and exhaustively annotated a multi-center dataset comprising digitized images from four European medical institutions. Using this dataset, we trained an ensemble of nnU-Net models for tumor cell detection in histopathology. For comparison, we also trained an ensemble of Faster R-CNN models. Finally, we conducted a reader study in the context of HDGC cell detection involving five pathologists, who annotated HDGC lesions to establish a human-based reference standard, enabling quantitative benchmarking of deep learning models.

## Results

### Annotated HDGC dataset supports development of robust AI models

We collected a total of 350 H&E-stained WSIs from 43 patients with a confirmed germline likely pathogenic/pathogenic variant of the *CDH1* gene, the most frequent but rare mutation underlying HDGC. Clinical metadata for the 43 patients is summarized in Table 1. The patients had undergone PTG between 2008 and 2023 at four centers: Radboud university medical center (Radboudumc, The Netherlands), the Netherlands Cancer Institute (NKI, The Netherlands), Unidade Local de Saúde São João (ULSSJ, Portugal), and the University of Padua (Italy). Histopathological review at each site identified tumor lesions, including intramucosal T1a lesions, intraepithelial *in-situ* and pagetoid lesions, for inclusion. The dataset was divided into three sets: a development set for model training and fine-tuning, an internal test set for performance evaluation on held-out cases, and an external test set to assess generalization to a different center. The Radboudumc and NKI slides were pooled and randomly split into development (80%) and internal test (20%) sets; ULSSJ data served as the external test set, and the single slide from Padua was held out for the reader study. All splits were performed at the patient level to ensure that no data from a single patient was included in more than one set.

**Table 1:** Clinical characteristics of included patients

	Min	Max	Median	Mean
**Full dataset (*n* = 43)**				

Age at gastrectomy (years)	18	64	38	36.0
Number of T1a lesions	0	227	15	25.5
Number of *in-situ* lesions	0	8	0	1.1
Number of pagetoid lesions	0	17	0	2.1

**Radboudumc (*n* = 22)**				

Age at gastrectomy (years)	19	56	30	33.4
Number of T1a lesions	0	131	11	21.6
Number of *in-situ* lesions	0	8	1	1.9
Number of pagetoid lesions	0	17	1	3.1

**NKI (*n* = 7)**				

Age at gastrectomy (years)	22	53	41	38.3
Number of T1a lesions	4	227	32	54.7
Number of *in-situ* lesions	0	0	0	0.0
Number of pagetoid lesions	0	6	0	0.9

**ULSSJ (*n* = 13)**				

Age at gastrectomy (years)	18	64	40	37.9
Number of T1a lesions	6	38	15	18.0
Number of *in-situ* lesions	0	2	0	0.3
Number of pagetoid lesions	0	9	0	1.3

**Padua (*n* = 1)**				

Age at gastrectomy (years)	53	53	53	53.0
Number of T1a lesions	3	3	3	3.0
Number of *in-situ* lesions	1	1	1	1.0
Number of pagetoid lesions	0	0	0	0.0

Overview of the characteristics of the included cases, presented for the full dataset and per center.

To establish a human performance benchmark for the cell detection task, we selected a subset of cases from the test sets for a reader study involving five pathologists. Sixteen tumor lesion images were selected from 10 patients in the test sets, and this set was supplemented with three additional images: one lesion from the Padua PTG, and two biopsies from HDGC patients at NKI and Radboudumc.

**Figure 1:**
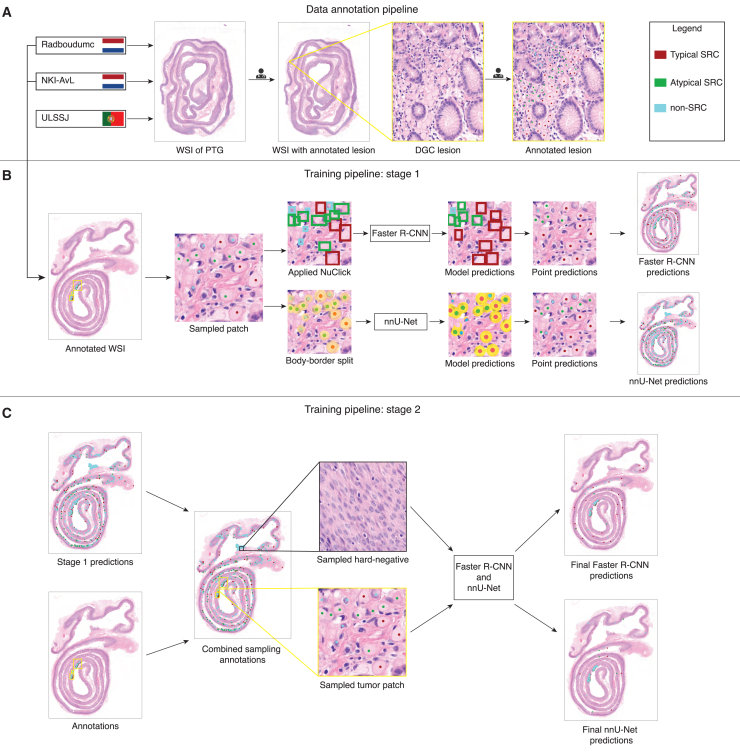
Annotated HDGC dataset and two-stage model development workflow Overview of the data annotation and two-stage model development methodology. In (A), we illustrate the multi-step annotation workflow, in which lesions are located and annotated in the WSIs, before annotating all tumor cells within those lesions. In (B), we illustrate stage one of the training pipeline, with the pre- and post-processing steps for nnU-Net and Faster R-CNN separated. In (C) we show how the model predictions after stage one are combined with the original annotations in the hard-negative mining procedure. During training, patches are subsequently sampled from both annotated tumor cells and hard-negative regions.

A total of 91,939 tumor cells were annotated across the WSIs using a four-step procedure. Tumor cells were categorized into three classes based on morphological criteria: 39% were typical signet ring cells (SRCs), 30% atypical SRCs, and 31% non-SRCs. Figure 1A illustrates the annotation procedure.

We employed a two-stage approach with 5-fold cross validation to train the nnU-Net and Faster R-CNN models. In the first stage, nnU-Net was trained on segmentation masks derived from point annotations, using a body-membrane split inspired by Swiderska-Chadaj and colleagues to help separate clustered cells at test time.^11^ Faster R-CNN was trained on bounding boxes generated via the pretrained NuClick algorithm.^26^ After stage one, we applied hard-negative mining to identify false positives, which were then incorporated into a second training stage to improve precision. This approach is depicted in Figure 1B and further outlined in section method details.

### Deep learning models match or exceed pathologist performance

We conducted a reader study on the Grand Challenge platform,^27^ where five pathologists (VA, LK, IG, FC, RP) independently annotated the images of the reader study dataset according to the definitions outlined in section method details. The trained models were applied to the same images, and their predictions were compared to the annotations from the five pathologists. This evaluation assessed model performance in relation to inter-pathologist agreement.

Inter-pathologist agreement was quantified by calculating pairwise F_1_ scores for pathologists’ annotations in the reader study dataset. F_1_ scores were computed separately for each cell type and then averaged across all cell types to yield an overall F_1_ score for each reader pair. The mean inter-pathologist scores are presented in Table 2, with the mean overall inter-pathologist F_1_ score calculated as 0.42 (95% CI: 0.41–0.44). When analyzed per cell type, the highest F_1_ score was observed for the typical SRCs, whereas substantially lower scores were noted for atypical SRCs and non-SRCs. Additionally, when considering agreement on detecting any tumor cell, irrespective of type, we found a higher F_1_ score of 0.74.

**Table 2:** Cell detection F_1_ scores from the reader study

	F_1_ overall	F_1_ typical SRC	F_1_ atypical SRC	F_1_ non-SRC	F_1_ any tumor cell
Inter-pathologist	0.42 (0.41–0.44)	0.70 (0.67–0.73)	0.34 (0.32–0.37)	0.23 (0.19–0.27)	0.74 (0.72–0.75)
nnU-Net	0.49 (0.46–0.51)	0.73 (0.70–0.76)	0.41 (0.37–0.44)	0.32 (0.27–0.38)	0.75 (0.73–0.77)
Faster R-CNN	0.41 (0.39–0.44)	0.67 (0.63–0.70)	0.28 (0.24–0.32)	0.29 (0.24–0.34)	0.67 (0.66–0.69)

Mean F_1_ scores and 95% confidence intervals from the reader study for the pathologists, nnU-Net model, and Faster R-CNN model, compared to the pathologists. F_1_ scores are presented averaged across all cell types, for each type individually, and finally for any tumor cell regardless of type.

**Figure 2:**
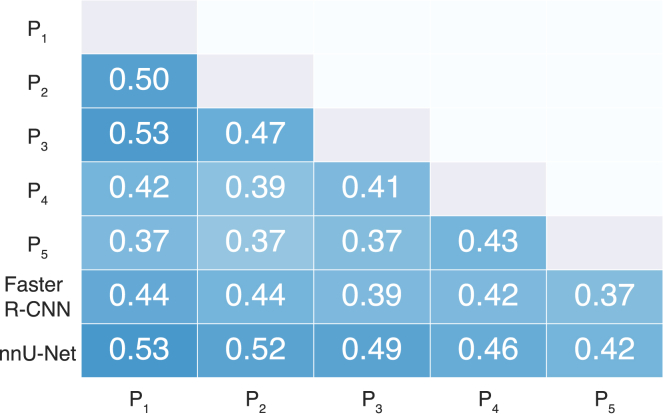
Pairwise AI-pathologist F_1_ scores in the reader study Overall F_1_ scores, computed as the mean over the entire reader study dataset, are presented pairwise between readers (P1-P5). Pairwise scores for AI model-reader pairs are shown in the last two rows.

**Figure 3:**
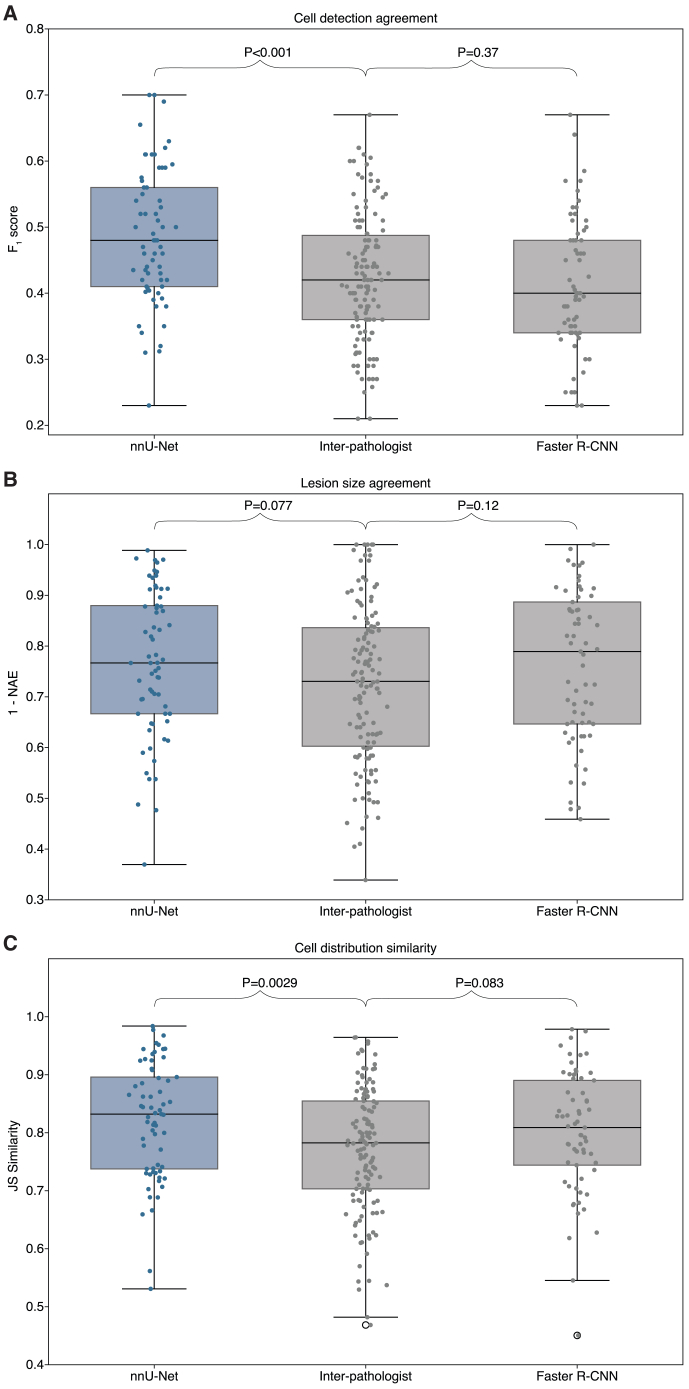
Inter-pathologist agreement and AI performance across reader study metrics Distributions of the pairwise reader study metrics at patient-level. The nnU-Net and Faster R-CNN distributions consist of pairwise comparisons of the model to a reader. The inter-pathologist distribution is determined by comparing pairs of pathologists. F_1_ scores are shown in (A), lesion size agreement in (B), and cell distribution similarity in (C). *p* values between distributions are calculated using the two-sided Wilcoxon rank-sum test.

The pairwise overall F_1_ scores, averaged over all images in the observer dataset, are illustrated in Figure 2. This matrix highlights the variability in agreement between different pairs of readers, as well as between pairs of readers and the AI models. As shown in Table 2, the nnU-Net model demonstrated significantly higher agreement with the readers than the readers achieved among themselves (*p* < 0.001). In contrast, the Faster R-CNN model showed no significant difference in agreement with the readers compared to inter-reader agreement (*p* = 0.37). The full distributions of the pairwise overall F_1_ scores per lesion are depicted in Figure 3.

**Figure 4:**
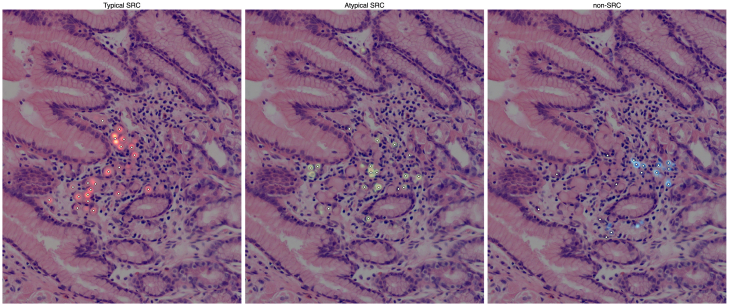
Reader study example with pathologist annotations and nnU-Net predictions Example case of the reader study, with reader annotations and predictions separated for each tumor cell type. The reader annotations are grouped and smoothed into heatmaps using a Gaussian kernel, and nnU-Net’s predictions are shown as white diamonds.

Finally, an example case from the reader study is shown in Figure 4. This case illustrates the readers’ annotations visualized as heatmaps, where brighter areas indicate cells with higher inter-reader agreement. Notably, nnU-Net predictions are predominantly concentrated in regions of higher agreement among readers, while areas of lower agreement correspond to fewer nnU-Net predictions.

It is worth noting that pathologists typically do not localize and classify tumor cells at a cellular level during diagnostics. Instead, they commonly assess DGC lesions by evaluating the overall lesion and estimating the amount of atypia on a larger scale. Following a similar approach, we also evaluated the models by calculating the total number of predicted tumor cells per lesion and analyzing the distribution of tumor cell types within each lesion. We used the normalized absolute error (NAE) to compare the total number of predicted cells per lesion with the reference annotations. To assess the similarity between the predicted and reference label distributions, we calculated the Jensen-Shannon (JS) similarity of the label distributions. The mean values for these metrics are given in Table 3. Notably, the values are substantially higher than the F_1_ scores measured during cell detection analysis, suggesting that predicting the lesion characteristics is an easier task for both pathologists and the AI models.

**Table 3:** Agreement on lesion characteristics from the reader study

	Agreement on tumor cell count (1 – NAE)	Agreement on cell type distribution (JS similarity)
Inter-pathologist	0.72 (0.70–0.75)	0.77 (0.75–0.79)
nnU-Net	0.77 (0.73–0.80)	0.82 (0.80–0.85)
Faster R-CNN	0.76 (0.72–0.80)	0.80 (0.77–0.83)

Mean 1-NAE values and JS similarities with 95% confidence intervals from the reader study for the pathologists, nnU-Net model, and Faster R-CNN model, compared to the pathologists. Agreement on the predicted tumor cell count is quantified by the 1 – NAE metric, while JS similarity measures agreement on the predicted tumor cell distribution.

The distributions of the pairwise metric values are shown in Figures 3B and 3C. Statistical analysis showed that, for the predicted number of tumor cells, neither deep learning model differed significantly in agreement with the readers compared to the level of agreement between the readers themselves (*p* = 0.077 for nnU-Net; *p* = 0.12 for Faster R-CNN). For the predicted cell type distributions, nnU-Net achieved significantly higher agreement with pathologists than the pathologists among themselves (*p* = 0.0029). In contrast, Faster R-CNN showed comparable agreement with the readers to that of the inter-reader benchmark, with no statistically significant difference (*p* = 0.083).

### nnU-net consistently outperforms Faster R-CNN across test sets

After establishing a baseline for pathologist performance on the reader study dataset and comparing the trained models to this benchmark, we evaluated the models on the full WSIs in the internal and external test sets. Predictions from each model were compared to the reference standard, and their performance metrics were analyzed to determine which model had the highest performance scores. This evaluation included F_1_ analysis at the full WSI level, as well as lesion-level analysis of tumor cell count agreement (1 – NAE) and cell type distribution agreement (JS similarity).

**Figure 5:**
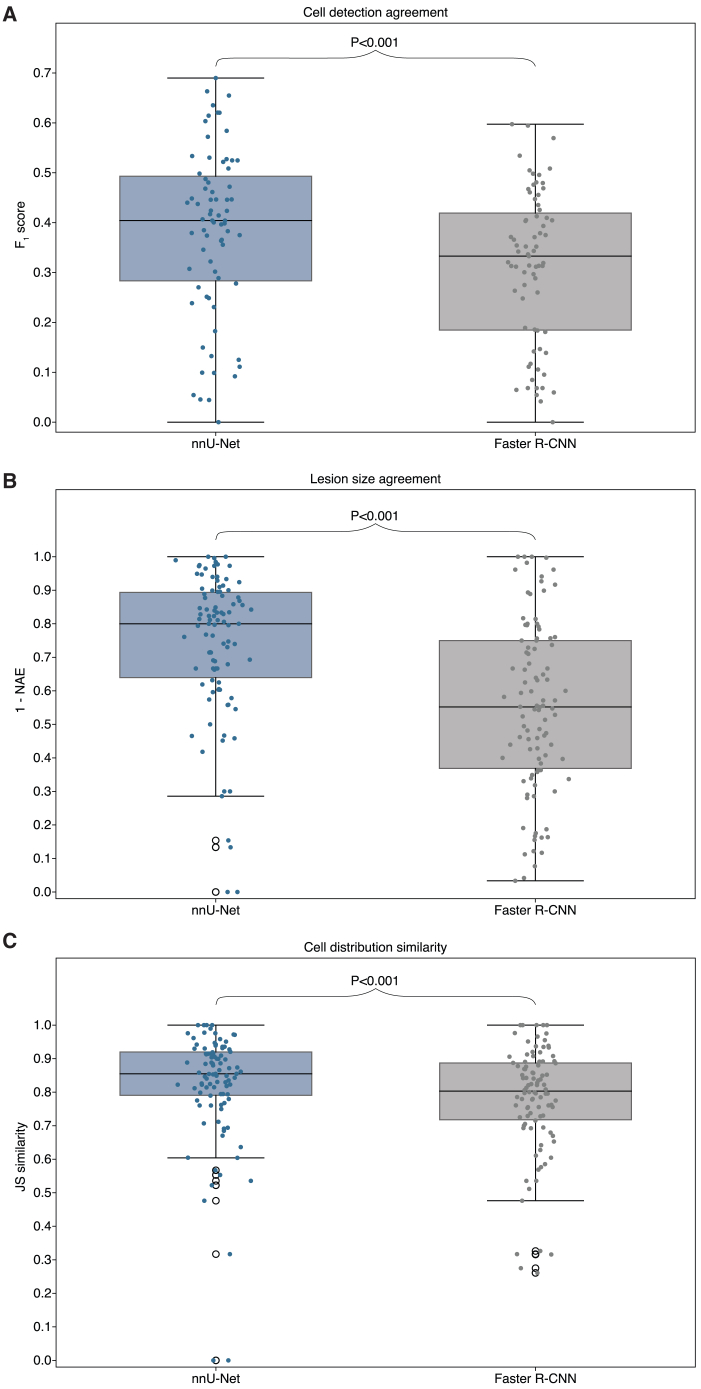
nnU-Net outperforms faster R-CNN on test set performance metrics Distributions of performance metrics on the test set for the nnU-Net and Faster R-CNN models compared to the reference standard. F_1_ scores are shown in (A), lesion size agreement in (B), and cell distribution similarity in (C). *p* values between the nnU-Net and Faster R-CNN results are calculated using the two-sided Wilcoxon signed-rank test.

Table 4 presents the mean values of the performance metrics, while Figure 5 illustrates their full distributions. Statistical analysis revealed that nnU-Net significantly outperformed Faster R-CNN across all three metrics (*p* < 0.001 for all metrics).

**Table 4:** AI model performance on the test set

	F_1_ overall (WSI-level)	1 – NAE (lesion-level)	JS similarity (lesion-level)
nnU-Net	0.38 (0.34–0.42)	0.74 (0.69–0.78)	0.82 (0.78–0.85)
Faster R-CNN	0.31 (0.28–0.35)	0.55 (0.50–0.60)	0.78 (0.74–0.80)

Mean F_1_ scores (WSI-level), 1-NAE values (lesion-level agreement on tumor cell count) and JS similarities (lesion-level agreement on cell type distribution) with 95% confidence intervals from the test set for the nnU-Net and Faster R-CNN models, compared to the reference standard annotations.

F_1_ scores were notably lower when evaluated on the full WSIs of the test set compared to the lesion-focused reader study. This discrepancy can likely be attributed to model errors in healthy tissue outside the lesion areas. To test this hypothesis, we conducted an additional F_1_ analysis focused exclusively on annotated lesions in the test set, ignoring regions outside these areas. In this lesion-level analysis, nnU-Net achieved an F_1_ score of 0.47 (95% CI: 0.44–0.50), while Faster R-CNN scored 0.41 (95% CI: 0.38–0.44). These scores are closer to the results from the reader study, and nnU-Net again significantly outperformed Faster R-CNN for this metric (*p* < 0.001).

### Deep learning models generalize well across internal and external test sets

**Figure 6:**
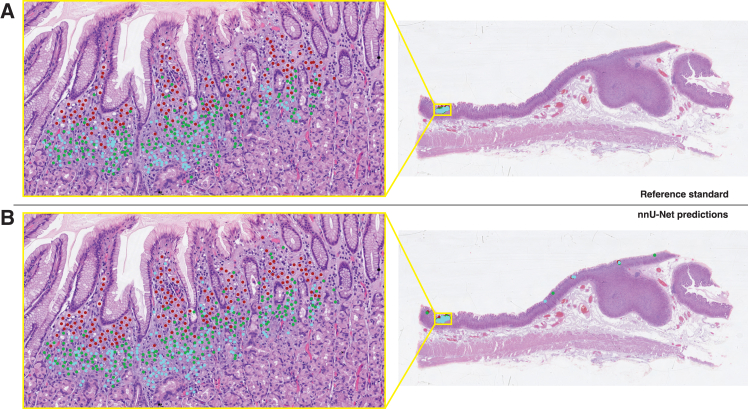
Accurate generalization of nnU-Net to external test set Illustration of the predictions of the trained nnU-Net models on a whole-slide image from the external test set. The reference standard is shown in figure (A), and the models’ predictions are shown in figure (B). Most model predictions are concentrated within the lesion area, with 496 predicted tumor cells located there, closely matching the reference standard (lesion-level F_1_ score of 0.70). Only 16 false-positive tumor cells were predicted in healthy tissue.

To evaluate the generalizability of the models, we compared their F_1_ scores on the internal and external test sets. The results are shown in Table 5. Statistical analysis revealed that neither model showed a significant difference in performance across the two sets (*p* = 0.14 for nnU-Net, *p* = 0.65 for Faster R-CNN). These results indicate consistent model performance across the internal and external test sets, suggesting model generalizability. Figure 6 shows the nnU-Net model predictions on a WSI from the test set, alongside the reference standard annotations for comparison. This figure highlights the remarkable similarity between the nnU-Net predictions and the reference standard. Additionally, the detection process on another WSI from the external test set is illustrated in Video S1.

**Table 5:** AI model performance on internal and external test sets

	F_1_ overall: internal	F_1_ overall: external
nnU-Net	0.35 (0.30–0.41)	0.43 (0.38–0.48)
Faster R-CNN	0.32 (0.28–0.37)	0.30 (0.26–0.35)

Mean F_1_ scores with 95% confidence intervals from the internal and external test sets separately, for the nnU-Net and Faster R-CNN models compared to the reference standard annotations.

Video S1. Detection of HDGC tumor cells in whole-slide image from external test set, related to Results Illustration of the detection process of the three tumor cell types of HDGC by the trained nnU-Net system. Typical signet ring cells are depicted in red, atypical signet ring cells in green, and non-signet ring tumor cells in blue. The nnU-Net system did not make any false positive predictions outside of the lesion area

## Discussion

Diagnostics of HDGC places a substantial burden on pathologists, as gastrectomies and endoscopic surveillance produce numerous slides requiring detailed histopathological analysis. Deep learning-based automated tools can assist pathologists by detecting tumor cells on WSIs from HDGC patients. Furthermore, understanding the distribution of different tumor cell types within DGC tumors can provide insights into tumor behavior. While there is an extensive body of work proposing promising methods for the detection of SRCs,^19^^,^^20^^,^^21^^,^^22^^,^^23^^,^^24^^,^^25^ none provide a multi-class detection model to address the composition of morphologically different tumor cells, including other poorly cohesive non-SRCs, present in DGC tumors. In this study, we addressed this gap by developing and evaluating models capable of detecting multiple tumor cell types in HDGC.

The main strengths of this study are 3-fold. Firstly, we compiled a multi-center dataset of 43 patients with confirmed pathogenic variants of the *CDH1* gene. In comparison, earlier work on AI for HDGC used a dataset of only seven patients.^28^ Our extensive dataset facilitated the development of robust models, as demonstrated by the consistent performance of the nnU-Net model across the internal and external test sets. Additionally, our annotation workflow produced 350 exhaustively annotated WSIs with 91,939 cell-level annotations—nearly five times the number of annotated tumor cells in the DigestPath2019 SRC dataset.^18^

Secondly, the reader study adds substantial value by providing context for the quantitative evaluation of the trained models. Five experienced pathologists evaluated 19 images from 13 distinct cases, resulting in an average of 1435 annotated tumor cells per pathologist. The resulting inter-pathologist agreement provides a valuable reference point for future research, enabling comparisons of tumor cell detection performance in early-stage DGC lesions. The nnU-Net model surpassed the human benchmark in tumor cell detection and in capturing the similarity of cell type distributions, while its performance in estimating lesion sizes was consistent with the inter-pathologist agreement range. These findings emphasize the significance of the reader study in evaluating model performance and highlight nnU-Net’s ability to match or even exceed pathologist-level performance in tumor cell detection and characterization.

Finally, we introduced a cell detection technique by training nnU-Net models and transforming the segmentation output into individual cell detections. When evaluating model performance on the test set, nnU-Net outperformed Faster R-CNN on all metrics, including tumor cell detection, cell distribution similarity, and lesion size estimation. This finding suggests that nnU-Net is not only suitable but may be the preferred approach for cell detection tasks in histopathology. Additionally, nnU-Net requires substantially less manual hyperparameter tuning than Faster R-CNN, making it easier to implement and more resource-efficient. Beyond detection, the segmentation output from nnU-Net offers potential for quantifying morphological features, such as cell size, circularity, and other geometric properties. As outlined by Da and others,^25^ tumor cell segmentation can be combined with morphological feature quantification to assess atypia. By applying their feature extraction approach to our nnU-Net-based segmentation, this method could be extended to the multi-class setting, enabling detailed characterization of different tumor cell types. This added capability further highlights nnU-Net’s versatility and value in both detection and downstream analysis.

The results of this study highlight the potential of nnU-Net models to transform the diagnostic workflow in histopathology of HDGC. With further development and validation on larger, more diverse datasets, these models could be integrated into the diagnostic process to assist pathologists by generating heatmaps or identifying suspected lesion locations in WSIs analyzed during PTG examinations. Beyond diagnostics, these models have potential applications in quantifying cell distributions and other tumor features identified during endoscopic surveillance, supporting a deeper understanding of DGC tumor behavior. In the future, these features may also contribute to defining DGC lesion subtypes, which could aid in stratifying patients for gastrectomy or endoscopic surveillance. Altogether, our study provides a strong foundation for advancing the diagnostics of HDGC through AI, with considerable translational potential for improving patient care.

### Limitations of the study

Despite its strengths, this study has several limitations that must be addressed before the developed nnU-Net models can be implemented in a diagnostic workflow. The models were trained exclusively on PTG specimens with early-stage DGC. While the morphology of DGC lesions is expected to be similar in biopsy specimens from early-stage cancers, the models should be fine-tuned and tested on a diverse set of biopsy images. Importantly, this should include advanced-stage DGC cases, where two key challenges are anticipated. First, the morphology of invasive tumor cells is likely to change with the depth of invasion, as reported by Humar and others,^6^ making these tumor cells appear different from those in early-stage lesions. Second, invasive tumor cells will be surrounded by tissue types that the model has either not encountered before or has seen only in non-tumorous contexts. Addressing these challenges will be crucial for extending the model’s applicability to more advanced DGC stages.

Additionally, although our dataset was multicentric, the external test set consisted of images from a single center. Validation on a larger, multicenter dataset is essential to further demonstrate the model’s generalizability before clinical implementation. Future work could aim to collect a more extensive dataset, incorporating a broader range of biopsies and resections spanning precursor lesions, early-stage cancers, and advanced DGC cases.

## Resource availability

### Lead contact

Requests for further information on software and resources should be directed to and will be fulfilled by the lead contact, Francesco Ciompi (francesco.ciompi@radboudumc.nl).

### Materials availability

This study did not generate new unique reagents.

### Data and code availability

• The image and annotation data in the Radboudumc dataset are available from the lead contact with a completed data transfer agreement.
• There are restrictions to the availability of image data from NKI, ULSSJ and the University of Padua, as sharing of these data are prohibited by data transfer agreements with the respective centers.
• All original code has been deposited at Zenodo and is publicly available at https://doi.org/10.5281/zenodo.15680064 as of the date of publication.
• Any additional information required to reanalyze the data reported in this paper is available from the lead contact upon request.

## Acknowledgments

This research was supported by an unrestricted grant of 10.13039/501100023452Stichting Hanarth Fonds, the Netherlands and the Netherlands Organization for Scientific Research (ZonMW 2023 clinical fellow 09032212110047).

## Author contributions

R.L.: conceptualization, data curation, formal analysis, investigation, methodology, software, validation, visualization, writing – original draft. V.A.: data curation, reader study participant, writing – review & editing. J.S.: software, validation, writing – review & editing. L.L.K.: data curation, reader study participant, writing – review & editing. I.G.: data curation, reader study participant, writing – review & editing. F.C.: data curation, reader study participant, writing – review & editing. R.S.v.d.P.: conceptualization, data curation, funding acquisition, reader Study participant, supervision, writing – review & editing. F. Ciompi: conceptualization, funding acquisition, supervision, writing – review & editing.

## Declaration of interests

F. Ciompi reports a relationship with TRIBVN Healthcare that includes: board membership and consulting or advisory. F. Ciompi reports a relationship with Aiosyn BV that includes: equity or stocks.

## Declaration of generative AI and AI-assisted technologies in the writing process

During the preparation of this work the authors used ChatGPT in order to improve language and readability. After using this tool, the authors reviewed and edited the content as needed and take full responsibility for the content of the publication.

## STAR★Methods

### Key resources table

**Table:** 

REAGENT or RESOURCE	SOURCE	IDENTIFIER
**Software and algorithms**		

nnUNet-for-pathology	Spronck et al.^17^	https://github.com/DIAGNijmegen/nnUNet-for-pathology/tree/nnunet_for_pathology_v2
Scikit-learn	Pedregosa et al.^29^	https://github.com/scikit-learn/scikit-learn
Weighted boxes fusion	Solovyev et al.^30^	https://github.com/ZFTurbo/Weighted-Boxes-Fusion
ASAP	Computational Pathology Group^31^	https://github.com/computationalpathologygroup/ASAP/tree/ASAP-2.1-(Nightly)
Detectron2	Wu et al.^32^	https://github.com/facebookresearch/detectron2
Source code	This paper	https://doi.org/10.5281/zenodo.15680064

**Other**		

Whole-slide images Radboudumc	Radboudumc	Available from the lead contact with a completed data transfer agreement
Whole-slide images NKI	NKI	Sharing prohibited by data transfer agreement
Whole-slide images ULSSJ	ULSSJ	Sharing prohibited by data transfer agreement
Whole-slide image University of Padua	University of Padua	Sharing prohibited by data transfer agreement

### Experimental model and study participant details

We collected data from *n* = 43 patients with a confirmed pathogenic germline variant of the *CDH1* gene, the most frequent but rare mutation underlying HDGC. We selected slides from 22 patients at Radboud university medical center (Radboudumc) in the Netherlands, seven patients at the Netherlands Cancer Institute (NKI) in the Netherlands, 13 patients at the Unidade Local de Saúde São João (ULSSJ) in Portugal, and one patient at the University of Padua in Italy, all of whom had undergone PTG during the periods 2011–2023, 2013–2022, 2008–2022, and 2022, respectively.

Of the 43 included patients, 63% were female and 37% were male. The proportion of female patients was 64% in the Radboudumc set, 57% in the NKI set, 69% in the ULSSJ set, and 0% in the Padua set. No analyses were performed to assess the influence of sex on study outcomes, as sex was not expected to affect histopathological tumor appearance or the performance of cell detection algorithms in this context.

In addition to sex, we collected data on age at gastrectomy and the number of tumor lesions identified during histopathological analysis, reported separately for pT1a, *in-situ*, and pagetoid lesions. An overview of these clinical variables is provided in Table 1.

We split the combined dataset into three distinct sets: 1) the development set, used to train and fine tune the deep learning models, 2) the internal test set, reserved to assess model performance on previously unseen data and to ensure the evaluation reflects generalization to new cases, and 3) the external test set, also unused during model training and tuning, aimed at evaluating the models’ robustness on cases originating from a different center. The Radboudumc and NKI datasets were combined into one set and then randomly split into development (80%) and internal test (20%) sets. The ULSSJ dataset was used as the external set. The slide from Padua was kept separate for the reader study. All data splits were made at the patient level, ensuring that no data from a single patient was included in more than one set.

This study was reviewed by the research ethics committee of Radboud university medical center (reference number 2022–13852) and was deemed to be in accordance with the applicable Dutch legislation such as the Medical Research Involving Human Subjects Act and the Medical Treatment Contracts Act.

### Method details

#### Data acquisition

Histopathological reviews were conducted (RP for Radboudumc, LK for NKI, FC and IG for ULSSJ, VA for University of Padua) to identify histology slides with tumor lesions, including intraepithelial *in-situ* and pagetoid lesions. This resulted in a total of 350 WSIs of H&E-stained slides: 249 from Radboudumc, 60 from NKI, 40 from ULSSJ and one from University of Padua. At Radboudumc, the slides had been previously scanned using a Pannoramic 1000 DX scanner (3DHistech, Hungary) at 40× magnification (0.24 μm/pixel). At NKI, the slides had also been scanned using a Pannoramic 1000 DX scanner (3DHistech, Hungary) at 40× magnification (0.24 μm/pixel). At ULSSJ, a NanoZoomer S360MD scanner (Hamamatsu, Japan) was used at 40× magnification (0.23 μm/pixel), and at University of Padua, a NanoZoomer-SQ (Hamamatsu, Japan) was used at 40× magnification (0.23 μm/pixel).

#### Reader study

We selected a subset of cases from the test sets for a reader study, in which five pathologists participated to set a reference standard for the cell detection task. We randomly selected three, two, and five patients from the Radboud, NKI, and ULSSJ test sets, respectively. From the WSIs of these patients, 16 images of tumor lesions were extracted. To add more variation to the reader study dataset, the set was supplemented by one tumor lesion from each of the following: 1) the PTG from the HDGC patient from Padua, 2) a biopsy from an HDGC patient from NKI, and 3) a biopsy from an HDGC patient from Radboudumc.

#### Data annotation

We exhaustively annotated all WSIs in the datasets using a four-step annotation procedure using the open-source software ASAP,^31^ involving two pathologists experienced with HDGC (RP, VA) and trained medical research assistants. First, a pathologist located and annotated all tumor lesions. Next, trained assistants placed point annotations near the center of each tumor cell and assigned class labels. The annotations were then reviewed and refined by one of the pathologists (VA), with final corrections made by the other pathologist (RP).

Tumor cells were classified intro three categories based on distinct morphological characteristics observed in this study and consistent with prior findings in the literature^33^^,^^34^: 1) typical SRCs, characterized by typical signet ring cell morphology as outlined in the 2019 consensus on poorly cohesive gastric carcinoma^8^; 2) atypical SRCs, which exhibit atypical morphological features such as atypical nuclear shape and size, often a smaller cell size compared to foveolar cells, and a higher nucleus-to-cytoplasm ratio than typical SRCs; and 3) non-SRCs, defined by a very high nucleus-to-cytoplasm ratio, scant cytoplasm, and increased nuclear atypia compared to atypical SRCs, possibly including evident nucleoli.

Using the outlined procedure and definitions, a total of 91,939 cell-level annotations were made, of which 39% were typical SRC, 30% were atypical SRC, and 31% were non-SRC. Figure 1A illustrates the data annotation procedure.

#### Model development

We employed a two-stage approach to train the nnU-Net and Faster R-CNN models, as depicted in Figure 1. The nnU-Net models were trained using the nnUNet-for-pathology framework,^17^ while the Faster R-CNN models were implemented with the Detectron2 library.^32^ Prior to training, a tissue-background segmentation algorithm was applied to generate tissue masks, ensuring that only tissue regions were analyzed.^35^

Training was performed on patches of 512 × 512 pixels, extracted from the WSIs in the development set at a resolution of 0.5 μm/pixel. We used a weighted sampling strategy, with 10% of patches sampled from healthy tissue outside of annotated lesions, and the remaining 90% sampled evenly from the three tumor cell types. Dynamic augmentations, including random flips, rotations, and HED color transformations,^36^ were applied to improve model generalization.

To prepare the sampled patches for nnU-Net, which requires ground truth labels for all pixels in an image, we transformed the point annotations into circular segmentation masks of fixed sizes for each tumor cell type. Combined with the tissue masks, this ensured complete pixel-wise labeling of the patches.

However, tumor cell segmentations often form connected clusters, complicating the identification of individual cells. To address this during training, we adopted an artificial separation of each cell into two concentric regions: an inner disk, referred to as the *cell body*, and a surrounding annulus, referred to as the *cell membrane*—an approach inspired by Swiderska-Chadaj and colleagues.^11^ During training, these regions were labeled separately, with the cell body as the target and the membrane serving as a border to help the model delineate adjacent cells. At test time, membrane predictions were removed, leaving only the disconnected cell body predictions, which were subsequently converted into individual cell point predictions. The radii of the cell body and membrane were based on estimates of the average size of each tumor cell type and are detailed in Table S1.

In contrast, Faster R-CNN requires bounding box annotations for training, which were generated by converting point annotations into bounding boxes using the pretrained NuClick algorithm.^26^

In the first stage, both models were trained using a 5-fold cross validation scheme with a fixed hyperparameter configuration, as detailed in Table S2. This stage was followed by a hard-negative mining procedure to reduce the number of false positive predictions. Specifically, we randomly selected 10% of slides from each fold in the development set, applied the trained models, and identified false positives—tumor cell predictions in non-tumor regions. These false positives, referred to as hard negatives, were added to the set of annotations for further training. For Faster R-CNN, false positives were grouped by their confidence scores and labeled accordingly.

In the second stage, the models were retrained using the updated annotations and revised sampling strategies. For nnU-Net, sampling weights were adjusted to allocate 20% of patches to each of the three tumor cell types, 10% to each hard-negative tumor cell type, and 10% to healthy tissue patches from regions without prior errors. Aside from these changes, the nnU-Net models were retrained with the same hyperparameter settings as in the first stage.

For Faster R-CNN, additional modifications were made in stage two. Sampling ratios between tumor and hard-negative regions were optimized, with higher-priority sampling given to hard-negative samples based on their confidence scores. Specifically, a hard-negative sample with score *x* was selected *x/y* times more frequently than one with score *y*. Additionally, we explored three backbone networks: ResNet-50 and ResNet-101 pretrained on the ImageNet dataset, and ResNet-50 pretrained on the Multi-Task Digital Pathology (MTDP) dataset.^37^ The optimal backbone and other hyperparameters were selected through tuning, with the tested parameters and search ranges listed in Table S3.

#### Validation studies

We validated the trained models using two approaches. Firstly, we conducted a reader study on the Grand Challenge platform,^27^ where five pathologists (VA, LK, IG, FC, RP) independently annotated the images of the reader study dataset according to the definitions outlined in section data annotation. The trained models were then applied to the same images, and their predictions were compared to the annotations provided by the five pathologists. This evaluation served to assess the models' performance in relation to the inter-pathologist agreement on these cases.

Secondly, the models were evaluated on the WSIs in the internal and external test sets. The models' predictions were compared to the reference annotations created during the dataset annotation procedure. This evaluation aimed to assess the models’ generalizability and compare the performance of the nnU-Net and Faster R-CNN models.

### Quantification and statistical analysis

The evaluation process involved patch-wise application of the models trained with 5-fold cross validation, postprocessing to refine predictions, and performance assessment using various metrics.

For the five nnU-Net models, we combined the predicted segmentation masks by assigning the class with the highest predicted probability to each pixel. The resulting masks underwent postprocessing: first, membrane predictions were treated as healthy tissue, separating the target cell masks. Next, we used connected component analysis from scikit-learn to identify centroids of the predicted cells,^29^ removing small components (radius < 2px) and close centroids (distance <10px) to reduce artifacts. This produced point predictions that were compared to the reference standard annotations.

To combine the predictions of the five Faster R-CNN models, we employed weighted boxes fusion.^30^ The fused box predictions were then converted to point predictions by extracting the center coordinates of each box.

We evaluated the models’ performance on the multi-class cell detection task using the F_1_ score, which balances precision and recall based on true positives, false positives, and false negatives at various confidence thresholds. A prediction was considered correct if it was within a 10 μm radius of an annotation of the same type. Additionally, to assess the models’ ability to capture lesion-level characteristics—reflecting how pathologists typically evaluate DGC lesions—we calculated the total number of predicted tumor cells per lesion and analyzed the distribution of predicted cell types. These predictions were compared to reference annotations using the normalized absolute error (NAE) for cell counts and the Jensen-Shannon (JS) similarity for cell type distributions.

For the reader study, all three metrics were calculated at the lesion level. The mean values and 95% confidence intervals for each metric were derived using the t-distribution. We employed the two-sided Wilcoxon rank-sum test to compare our models’ performance against the human benchmark. For each lesion, we calculated pairwise metric scores between each model and each reader and compared these scores to the inter-pathologist scores. The null hypothesis assumed no difference between model performance and the pathologist benchmark. A *p*-value smaller than 0.05 indicated a statistically significant deviation from the reader-established benchmark. In cases of significant differences, the 95% confidence intervals were used to assess whether the model outperformed or underperformed relative to the benchmark.

For the model evaluation on the test set, metrics were calculated using bootstrapping with 1000 iterations, and 95% confidence intervals (CIs) were derived from the 2.5% and 97.5% percentiles of the resulting distributions. Statistical significance was assessed by comparing the metrics of the two models using two-sided Wilcoxon signed-rank tests, with a *p*-value of less than 0.05 considered significant. When a significant difference was identified, the direction of the difference was determined by analyzing the 95% confidence intervals.
