# Supplementary material for: Deep learning for multiclass tumor cell detection in histopathology slides of hereditary diffuse gastric cancer
Source: iScience. 2025 Jul 5;28(8):113064. doi: 10.1016/j.isci.2025.113064 (PMC12303045; doi:10.1016/j.isci.2025.113064)
Supplement: Document S1. Tables S1–S3 [file mmc1.pdf]

iScience, Volume *28*

Supplemental information

Deep learning for multiclass tumor cell detection in histopathology slides of hereditary diffuse gastric cancer

Robin Lomans, Valentina Angerilli, Joey Spronck, Liudmila L. Kodach, Irene Gullo, Fátima Carneiro, Rachel S. van der Post, and Francesco Ciompi

Table S1: Radii of the artificial cell body and cell membrane annotation masks used during nnU-Net training.

	Cell body thickness (μm)	***Cell membrane thickness (μm)***
Typical SRC	3.75	6.25
Atypical SRC	3.75	3.75
non-SRC	3	3.25

Table S1: The radii used when converting cell point annotations to circular cell segmentation masks for nnU-Net training.

Table S2: Hyperparameter configuration for stage one model training.

	Faster R-CNN	***nnU-Net***
Model architecture	ResNet-50 (ImageNet pretrained [S1])	U-Net-like
Batch size	16	8
Optimizer	SGD	SGD
Learning rate	0.001	0.01
Weight decay	None	$3*10^{-5}$

Table S2: Hyperparameter configuration used in the training stage of the Faster R-CNN and nnU-Net models.

Table S3: Tuned hyperparameters in stage two of training Faster R-CNN.

Parameter	***(Range of) values tested***
Initial learning rate	[0.005, 0.05]
Learning rate decay iterations	{0, 40000}
Weight decay	[0.0001, 0.0005]
Warmup iterations	[50, 500]
Positive-negative sampling ratio	{2, 4}

Table S3: An overview of the hyperparameters tuned during stage two of training Faster R-CNN. Ranges of values are indicated by square brackets [a, b], while discrete tested values are denoted by curly brackets {a, b}.

Supplemental references

S1. Russakovsky, O., Deng, J., Su, H., Krause, J., Satheesh, S., Ma, S., Huang, Z., Karpathy, A., Khosla, A., Bernstein, M., et al. (2015). ImageNet Large Scale Visual Recognition Challenge. Int J Comput Vis 115, 211–252. https://doi.org/10.1007/s11263-015-0816-y.
